# A Qualitative Examination of the Detroit Community Food Response to COVID-19

**DOI:** 10.3390/nu15133047

**Published:** 2023-07-06

**Authors:** Michelle M. Gilleran, Aeneas O. Koosis, Alex B. Hill, Alyssa W. Beavers

**Affiliations:** 1 Department of Nutrition and Food Science, Wayne State University, Science Hall 410 W Warren, Detroit, MI 48201, USA; gs6226@wayne.edu (M.M.G.); hi4418@wayne.edu (A.O.K.); 2 Department of Urban Studies and Planning, Wayne State University, Faculty/Administration Bldg 656 W. Kirby, Detroit, MI 48201, USA; alexbhill@wayne.edu

**Keywords:** COVID-19, food assistance, emergency food

## Abstract

The COVID-19 pandemic increased the need for food assistance due to surging unemployment, the closure of in-person schooling, and other factors. This posed a historic challenge to organizations that address food insecurity: meeting the surging need for food while minimizing COVID-19 transmission. This study aimed to identify how food insecurity program operations changed during the pandemic and to examine the facilitators/successes and barriers/challenges to operations. Semi-structured interviews were conducted with staff at 13 organizations involved in addressing food insecurity in Detroit during the pandemic. Interviews were coded by two coders, summarized, and then used to create matrices and concept map displays for each organization. We found that nearly all programs changed to a contactless food distribution format, and most programs experienced an increase in demand for food. Common successes/facilitators included keeping clients and staff safe from COVID-19 and waivers that eased program rules. Common challenges/barriers included the increased need for labor and food. Lack of funding was a barrier for some organizations, and others that experienced an increase in funding reported that it facilitated their work. This research identified the needs of programs addressing food insecurity during the COVID-19 pandemic, which can inform future disaster planning.

## 1. Introduction 

Food insecurity is defined as “limited or uncertain availability of nutritionally adequate and safe foods or limited or uncertain ability to acquire acceptable foods in socially acceptable ways” [1]. It is a complex, multifactorial public health issue that can occur at both the individual and household levels. Additionally, food insecurity is associated with negative health outcomes, such as type 2 diabetes mellitus, hypertension, and hyperlipidemia [2,3,4]. While food insecurity often occurs in low-income households [5], income is not the only determinant of food insecurity. Food insecurity can occur when any of its four dimensions are compromised: food access, food availability, food utilization, or stability [6]. 

During the COVID-19 pandemic, the efforts necessary for reducing the spread of the virus had the unfortunate side effect of disrupting each dimension of food security [7]. For example, meat-packing plants closed during COVID-19 outbreaks among workers, which reduced the available supply of meat, and food availability was also reduced in stores due to consumer panic buying [8]. The closure of schools and daycares reduced children’s access to nutritious food, while the closure of nonessential businesses resulted in massive job losses that reduced financial access to food. In March 2020, the U.S. Bureau of Labor Statistics reported an unemployment rate of 14.7%, a rate that more than tripled from 4.4% in February 2020 [9]. This unprecedented rise in unemployment, coupled with an increase in the food at home consumer price index [10], had devastating effects on U.S. household food security. The Census Household Pulse Survey found that approximately 9.1% of adults in the United States reported there was either sometimes or often not enough to eat in the last seven days in April 2020, and by December 2020, this figure jumped to 13.7% [11]. These findings indicate that maintaining food security during the first year of the COVID-19 pandemic was a pressing and persistent challenge for many households in the United States. 

The charitable feeding system and federal nutrition assistance programs were called upon to play a vital role in addressing food insecurity during these challenging times [12]. The charitable feeding system in the U.S. primarily consists of food banks and their partnering food pantries. Food banks are regional organizations that take in large volumes of food, and food pantries distribute that food to people in need. Feeding America, an umbrella organization with member food banks across the U.S. [13], served 55% more people in 2020 than the year prior [14]. The United States Department of Agriculture (USDA) increased allotments and loosened eligibility requirements for the Supplemental Nutrition Assistance Program (SNAP), the U.S. government’s largest food assistance program [15]. In February 2020, 37 million Americans participated in SNAP, and by June 2020, this number rose to 43 million [16]. 

The U.S. government also launched two new federal food assistance programs during the pandemic: the Pandemic-Electronic Benefits Transfer (P-EBT) and the Farmers to Families Food Box Program (FFFBP) [17]. P-EBT provided qualifying households with money for groceries equivalent to the value of school meals missed due to school closures. Through the FFFBP, the USDA purchased food products from producers and donated them to charitable organizations for distribution to households in need. Although utilizing federal nutrition assistance programs and charitable food are common strategies used by many households experiencing food insecurity [12], the impacts of the pandemic on the food supply chain made this reliance challenging. For example, due to consumer panic buying and reduced meat in the supply chain, food procurement challenges for charitable food organizations emerged. This was particularly true for meat and shelf-stable items [18]. Additionally, as the demand for charitable food skyrocketed, there were news reports of incredibly long lines at food distributions, and organizations sometimes ran out of food [19]. 

To identify best practices and recommendations for food programs during future times of increased food insecurity, it is essential to examine how these programs functioned during the COVID-19 pandemic. There are currently only a small number of studies that have examined how safety net programs in the United States responded to the increase in demand for food during the pandemic. The few existing studies focus on non-governmental charitable food assistance programs, such as food pantries and food banks. These few studies found that these organizations made operational changes to labor/volunteers, the food distribution process, and the amount of food distributed [20,21,22]. Additionally, Jablonski et al. identified indicators of effective programs, such as cross-sector collaborations and addressing gaps in service to high-risk populations [23]. However, more research on a wider variety of food assistance programs is needed to understand how food insecurity was addressed during the pandemic. In our study, we examine how a variety of food assistance programs responded to the increase in food insecurity during COVID-19, identify the perceived challenges and successes of these efforts, and identify perceived gaps in who is being served by food organizations and programs. 

## 2. Methods 

### 2.1. Data Collection 

This qualitative study was conducted through semi-structured interviews conducted in 2021 with the staff of organizations involved in addressing food insecurity in Detroit, Michigan, during the COVID-19 pandemic. Detroit, Michigan, is a city disproportionately impacted by poverty and food insecurity [24] and was also disproportionately burdened with COVID-19 cases [25]. To be eligible for an interview, organizations must have performed work to address food insecurity in Detroit, Michigan, during the COVID-19 pandemic. This work included providing free food, addressing food access, or participating in advocacy activities. Eligible organizations were identified by the United Way of Southeastern Michigan, a local chapter of a large national U.S. nonprofit organization whose focus areas include helping the community in meeting basic needs, including food. Organizations were selected by purposive sampling to obtain a diverse representation of local efforts aimed at addressing food insecurity, including both governmental and non-governmental efforts and programs that served varying target audiences. Selected organizations were contacted via email to request an interview with a member of their staff. Interviews were conducted online via Zoom and lasted 60–90 min. Interviewees provided verbal consent for the research and consent to be recorded prior to recording the interviews. Interviews were audio recorded, transcribed, and transcriptions were checked for accuracy.

The semi-structured interview guide was developed by the authors and informed by feedback from staff at United Way of Southeastern Michigan due to their extensive knowledge of both federal and charitable food assistance programs. The interview guide contained broad main questions coupled with probes and follow-up questions to elicit more detail [26]. The interviewees were asked to describe their organization’s programs before the COVID-19 pandemic, identify how the programs changed when the pandemic began, and describe any new programs that started during the pandemic. The remaining questions were designed to obtain the participants’ perspectives regarding what went well and where these programs struggled during the pandemic. For example, participants were asked “What are the biggest successes you’ve seen since the pandemic” and “What have been the biggest challenges to your work”. To identify if there are gaps in who is being served by these Detroit food organizations and programs, participants were asked “Who do you think you are doing the best job reaching” and “Is there anyone you think is not being adequately reached since COVID?” This research was approved by the Wayne State University Institutional Review Board (IRB), protocol number 20-12-3009. 

### 2.2. Analysis 

A combination inductive–deductive approach was used for coding. A preliminary codebook was developed using deductive codes that aligned with the research questions and was iteratively modified inductively during analysis [27]. For the first phase of coding, two researchers independently coded each interview transcript and then met weekly via Zoom conference calls to discuss similarities and differences in coding. When coding differences were found, the two coders reviewed the codebook and the interview transcript to reach coding consensus. During these meetings, the codes and definitions were refined as needed (e.g., editing code definitions, combining similar codes, and adding new codes). For the second phase of coding, one coder developed and applied more detailed subcodes from the broader first-phase codes. For example, under the broader code of “successes”, subcodes of “COVID-19 safety” and “large number of people reached” were created. For each organization, coded data were summarized and put into a conceptually clustered display [27]. These displays included summaries and representative quotes under each of the following categories: 1. a description of each food program before COVID-19, 2. the changes each program underwent when the pandemic began, 3. challenges participants reported, 4. successes participants reported, and 5. who was served well and who was not adequately reached. The displays were reviewed to identify similar patterns across cases [27].

## 3. Results

### 3.1. Organization Characteristics 

The researchers contacted 15 organizations to request an interview. Representatives from 13 of the 15 organizations responded to the request, and a total of 14 interviews were conducted with representatives from 13 different organizations. Of the 13 organizations interviewed, 11 had programs that addressed food insecurity by providing food prior to the pandemic. During the COVID-19 pandemic, 12 out of the 13 organizations provided food. The characteristics of each organization, including the type of organization, a brief description of food program(s), and program audience prior to the pandemic are listed in Table 1. 

### 3.2. Interview Findings 

First, we present how existing food programs changed after the onset of the pandemic and then describe new food programs developed during the pandemic. Following that are the results regarding the organization representatives’ perceived challenges and successes of these efforts and the perceived gaps in who is being reached by these organizations. 

### 3.3. Program Changes during the Pandemic 

Each of the food programs operated by the organizations underwent changes to reduce the risk of spreading COVID-19. Many of the changes reported fell into the following categories: changes in the amount of food distributed and changes in the food distribution model and format. Below, these changes are presented by program type. 

#### 3.3.1. Food Banks and Food Pantries

Three of the organizations interviewed provided food to the community through food pantries, and two were food banks that operated some food pantries themselves but also collaborated with other partner organizations to distribute food. The two food banks distributed 10 and 40% more food, respectively, in 2020 compared to 2019, despite the closure of many of the food pantries operated by their partner agencies. In order to compensate for the closure of partner pantries, while also keeping up with the surge in food need, the food banks began distributing much more food themselves as opposed to through their partners. One food bank representative reported developing what they termed “supersites”, food distributions that could serve over 700 families, much larger than a typical food pantry that served under 200 families. The other food bank reported developing mobile pantries that they operated themselves and were specifically targeted to areas where children and seniors were located. Both representatives reported that the increased volume of food required them to rent a larger facility for food storage and obtain more food trucks to transport food. 

Prior to the pandemic, nearly all of the food pantries operated by interviewed organizations used a client-choice format where clients selected their own food from a variety of options offered. To protect both the staff and clients from COVID-19 exposure, almost all the organizations shifted to pre-packed boxes that could be picked up through a curbside model. This allowed them to efficiently increase their distribution during the pandemic. One senior-serving organization also increased home deliveries for their pantry program during the pandemic. While the home-delivery option was offered prior to the pandemic, it became especially vital for seniors who did not feel comfortable coming to the curbside pick-up sites: “[There was] just a general fear of the virus. For seniors who were really at risk, going anywhere could be a dangerous proposition. If someone needed a delivery, we didn’t ask any questions”. 

While the pre-packed boxes distributed through curbside pickup were beneficial for protecting from COVID-19 exposure, several interviewees stressed the importance of returning to a client-choice pantry model once it was safe to do so. As one representative stated:

*I think that in my opinion it [client-choice pantry] was the goal for us …The word was choice pantry. How are you going to give me lima beans if I don’t want them? Why should I just have a box? I think that you want to give the people personal choice on what they want. It just makes them feel better to have a choice. If not, you’re wasting that food in general.*

#### 3.3.2. Prepared Meal Programs

Prior to the pandemic, six of the interviewed organizations operated prepared meal programs that provided one meal per person for on-site consumption; five of these programs were solely for youth; and the other was solely for seniors aged 60+. During the pandemic, two of the youth programs provided groceries as opposed to prepared meals, while three youth programs shifted to distributing multiple meals at once through curbside pick-up. These food pick-ups also served as a touch point to provide other services. As the interviewee stated, “it was like a full drive-through system, pick up the food, get your computer repair, and then you can go right back out”. In addition to the curbside model, three of the five youth-serving meal programs offered a home-delivery option. The interviewees from these organizations reported that the delivery option was primarily used for “medically fragile” students or families that had transportation challenges. As one interviewee stated, “We were able to fill in the gap, respond quickly … A lot of them, they struggle with transportation”. 

There was a large variation in how the reach of these youth food programs changed from pre-pandemic to during the pandemic. The two organizations that operate summer meal programs (programs that provide prepared meals during the summer months when children are not in school) increased their reach: one reported doubling the number of meals served, while the other provided nearly 1.4 million meals in 2020, up from an estimated 80,000 meals in 2019. Representatives from both of these organizations attributed the increase to a change in program format. Pre-pandemic, both organizations provided prepared meals that were required to be eaten on site; one of the organizations transitioned to a to-go meal format, while the other provided groceries to prepare meals at home.

In contrast, the school district representatives reported a decrease in school meal program participation. Like one of the summer meal programs, both school meal programs converted to a to-go curbside pickup format. One of the school district representatives partially attributed the reduction in participation to the P-EBT program: the benefits reduced the need for some families to pick up school meals, especially since meals were only provided for the children in the household as opposed to the whole family. When discussing this reduction, the representative stated, “[Because of P-EBT], people have the money to buy groceries… They don’t need to come pick up food from us to fill their refrigerator when they have money to go buy whatever they want to cook”. Additionally, both school district representatives reported that barriers to accessing the to-go school meals, such as lack of reliable transportation or the limited times the meals were offered, likely reduced program participation. As one interviewee said, “If I’m a student who will normally eat school lunch and mom is normally at work when it’s lunchtime for me, clearly, I’m not going to be allowed to just walk up to school and pick up food, so where does my food come from?” 

Lastly, one organization offered prepared meals to seniors (adults aged 60+) through two separate programs: a congregate meal program that provided prepared meals for on-site consumption at locations throughout the city and a home-delivered meal program that offered clients a choice between a hot meal delivered five days a week or a five-pack of frozen meals delivered once weekly. During the pandemic, the hot meal options were discontinued for both programs. All clients participating in the home-delivered meal program received the five-pack of frozen meals once weekly, while the congregate meal program changed to a curbside pick-up of five-packs of frozen meals (the same frozen meals they provided their home-delivered meal clients). This transition was made to reduce the number of contact points, thus reducing the risk of COVID-19 exposure for both the clients and staff. The interviewee reported that making the shift entirely to frozen meals helped the organization expand their reach, stating that “[frozen meals] allowed us an opportunity to expand how many people we could serve. That’s how we were able to ramp up so quickly and triple the reach”. Additionally, this senior-serving organization was able to offer clients other items beyond food, such as bottled water, face masks, and hand sanitizer, with the interviewee stating that “That’s not what we normally do … We were doing additional things to help people”.

### 3.4. Development of New Food Programs 

In addition to existing programs changing, 8 of the 13 organizations developed new food programs to meet the new challenges that arose during the pandemic, namely food waste caused by food supply chain disruptions and reducing the spread of COVID-19. 

#### 3.4.1. Programs to Reduce COVID-19 Spread

Some of the new programs were developed specifically to reduce the risk of COVID exposure or spread. For example, a home-delivered food program was developed by a government agency to help ensure that people who had COVID-19 did not need to leave their home to obtain food. Through collaboration with the city health department, residents who tested positive for COVID-19 were referred to the program if they indicated they needed support receiving food while quarantining. Another home-delivery program was developed to enhance the safety of food distribution, especially for older adults more susceptible to COVID-19 infection. This program delivered boxes containing shelf-stable food items that could be used to make quick meals at home. An interviewee described what precipitated the development of this program:

*[The boxes] were easier for some of our senior partners to distribute because we could drop them at the senior housing place and the manager could set them in front of somebody’s door. This [program] started because we had mobile pantries that were serving senior sites, and they [the seniors] were congregating downstairs and that’s just not okay. What the benefit [of the boxes] was, it was a safer delivery.*

Another interviewed organization reported that these boxes were especially useful for seniors who did not want to leave home due to the fear of COVID-19. 

While not directly providing food, a food policy and advocacy organization developed a grocery store safety program. This program focused on safety practices at grocery stores so that not only the public could feel safe to shop but also the staff at grocery stores felt safe and protected from COVID-19 exposure. By partnering with local organizations, this program provided grocery and other food stores with face masks, gloves, hand sanitizers, and social distancing stickers and signs to be placed throughout the store. Additionally, this program advocated for food workers to be recognized as essential workers in order to become eligible for COVID-19 vaccines earlier. When discussing the successes of this program, the representative stated:

*That was really tangible—to be able to go to my local grocery store and see the signage, to see the stickers, to see people able to wear a mask, and to know that we really did help people stay a little bit safer. Another win was it wasn’t just us, but we were one of the people, like I said, advocating for grocery store workers to be able to get vaccinated and to see them included in essential workers.*

#### 3.4.2. Programs to Reduce Food Waste

Other new programs were developed to make use of food that would otherwise go to waste. One organization was approached by an airline food service company that had a surplus of perishable food early in the pandemic when air travel was greatly reduced. The airline put together meals that the organization distributed to seniors. While this was a short-term program that was discontinued when the airline used up their food surplus, it was mutually beneficial to both the food service company and the organization. This interviewee stated, “What [the program] was doing was, they [the food service company] were working to help individuals continue working during that period of time. What they were able to do was provide meals to individual groups like us for free, just to use up all the stock that they had available”. 

Another food waste reduction program was developed by an interviewed nonprofit organization that, pre-pandemic, focused on providing education focused on food waste reduction and advocacy efforts. Due to the massive food supply chain disruptions that arose when the pandemic started, this organization shifted much of their efforts during COVID-19 to rescuing surplus food from grocery stores, restaurants, farmers, and food distributors. The interviewee reported that despite several other local organizations rescuing food, there was still a large amount of food going to waste, and there were barriers to distributing such a large amount of surplus fresh food to the community: “The community can’t always absorb it quickly enough, especially if it’s fresh… You have some food pantries that are taking in food, and either they can’t store it or they can’t get it out fast enough…”. They worked with another local organization to distribute this rescued food and also used it to prepare meals and distribute them to the community.

### 3.5. Perceived Organization Success and/or Facilitators during the COVID-19 Pandemic 

While some organizations reported that the food program changes during the pandemic were exhausting and labor-intensive, all the organizations unanimously agreed that the successes achieved during the pandemic made their efforts worthwhile. 

#### 3.5.1. Staff/Organization Resilience 

Despite demand at food assistance organizations skyrocketing, several interviewees reported the organization and staff demonstrated resilience in adapting to the changes and challenges brought on by COVID-19. This was evidenced by many interviewees expressing how the staff were willing and eager to perform the additional work required to maintain food operations. Interviewees said things such as “we were all hands on deck” and “everybody did an awesome job of really pulling together and doing whatever it was we had to do to get food distributed”. Many interviewees reported that this resiliency stayed constant even when staff had to work longer hours and/or during atypical hours to distribute meals (i.e., late nights or holidays) and when staff were reassigned from their normal positions to work in food distribution areas. One interviewee stated, “The biggest successes, I would say, completely changing the model of how we provide services literally within a week, and not skipping a beat, and still delivering meals to all the people that we were serving the week prior. That was huge”. Another interviewee stated:

*In those early days, people were calling each other nine o’clock on a Saturday night, Sunday afternoon. There was a lack of time, I guess. You could think of it as that is a bad thing, but what I think of it as, is a commitment to the effort, which I found really just inspiring. I found it uplifting. Of course, it was also eventually exhausting, but, I think that is a real thing to call out, is exhaustion and fatigue on the part of the nonprofits and their staff, because it just ground on for so long.*

Some organizations reported that this resilience contributed to the increased amount of food the organization was able to distribute throughout the pandemic. Eight organizations reported that a major success was the large number of people they were able to reach and/or the amount of food they were able to distribute. These interviewees displayed a sense of pride and gratitude for the effort the staff, volunteers, and organization contributed to continue distributing food. When describing this success, interviewees said things like “I’m just glad that we’re able to serve people, and not turn anybody away” and “If you showed up here and needed help, we gave you help”. 

#### 3.5.2. Program Waivers 

For organizations that operated federally regulated food programs, program waivers that relaxed requirements contributed to the organizations’ success during the pandemic. Pre-pandemic, most of the prepared meal programs (congregate senior meals, school meals, and summer feeding programs for youth) were mandated to have the meals eaten on site, termed congregate feeding. During the pandemic, a federal waiver permitted non-congregate feeding, allowing food to be packaged to-go. For both school meals and summer feeding programs, another waiver not only permitted parents and/or guardians to pick up food without the student present but also allowed for multiple days of meals to be picked up at once. An additional waiver permitted school meals to be distributed to any school-aged child, not just those that attended that school. For another program, a waiver allowed for organization staff to sign paperwork on behalf of their clients. Interviewees reported that these waivers created flexibility that allowed them to serve more people compared to pre-COVID-19. A representative from a government agency stated:

*All of that [the waivers] was a massive game-changer for the way that we had thought about doing that type of meal distribution. Really, I think had those waivers not been in place, certainly, we would not have been able to provide the level of service we did and have.*

#### 3.5.3. Sufficient Funding 

A further success highlighted by several organizations was the funding made accessible from the federal government or through generous donations. Interviewees reported that additional funding aided in purchasing food, materials for food packaging (boxes, to-go containers, bags, etc.), program development, food deliveries, and COVID-19 safety at stores and food distribution sites (signs, hand sanitizer, gloves, and masks). One senior-serving organization reported they were able to double the number of clients they serve primarily due to adequate funding. This organization typically has a large waitlist, but the interviewee reported that “If the funding were to stay at the levels that it was at the beginning of the pandemic throughout the year, we would be able to serve everyone. There would be no waitlist”. Another example of sufficient funding came from a food bank that typically does not purchase its food but rather obtains the food they distribute through food rescue. They noted that during the pandemic, the vast amount of funding and donations received allowed them to purchase additional food for distribution. 

#### 3.5.4. COVID-19 Safety 

Providing food while ensuring the organization’s staff and community were safe from COVID-19 was a common success reported by interviewees. One interviewee stated, “We fed a lot of people, but we also did it in a very safe way … there’s not one case that’s traceable back to [the organization]”. Interviewees reported implementing a variety of measures to protect the staff and community from viral exposure, such as distributing personal protective equipment (PPE) including masks and gloves, employing sanitization practices to keep the facility and food delivery trucks clean, and performing temperature checks before staff entered the facility. Furthermore, some organizations reported reducing staff, volunteers, and collaborations that brought personnel into the facility. One school representative stated, “We had a moratorium on that [new partnerships] because of COVID-19. People were coming in from outside who could potentially be carrying the virus. Just limiting contact points. Limiting exposure points”.

While most organizations deemed COVID-19 safety measures a success, a few expressed challenges in procuring PPE or coping with the added expense. Although the majority reported high PPE compliance from staff and community members, a farmers’ market organization encountered difficulties in getting some food vendors to adhere to face mask requirements, which led to the organization asking non-compliant food vendors to leave, upholding the COVID-19 safety guidelines. 

#### 3.5.5. Collaborations 

Interviewees reported that both old and new collaborations helped their organization in a number of ways, such as directly providing food, funding for food, and/or food distribution materials and supplies, PPE, and social distancing signs for COVID-19 safety; transportation to deliver food to residents’ homes; and providing space to use for food storage, preparation, and/or distribution. Some organizations reported that communicating with collaborators helped their organization learn how to operate their food programs safely during a time with so many unknowns. Furthermore, some interviewees observed that such communication enabled organizations to identify the extent of their reach and gaps in serving the community. Simultaneously, it increased awareness within the organization about the variety of food assistance programs accessible to Detroit residents in need. 

Of the 13 organizations interviewed, 12 started new collaborations since the start of the COVID-19 pandemic. Interviewees reported that the new collaborations not only contributed to success during the pandemic but may also be beneficial beyond the pandemic. A representative from a food waste reduction organization stated:

*We tend to attract and work with people who are really generous and just the roll up your sleeves and get it done…It’s just like, ‘Whoever’s got the best idea, let’s just go do that and let’s support each other.’ … We’re doing this for the community. We’re doing this for the earth. We’re doing it for the right reasons.*

#### 3.5.6. Sufficient Food Supply 

Despite increased demand for food and the widespread food supply issues during the pandemic, some organizations reported they were able to successfully meet the demand. As a school representative reported, “There’s never been a point where we were just out of food. We’ve had to make more substitutions this year than probably the entirety of my career here at [organization] because of it, but it’s still- it’s still working”. 

Some organizations reported that an important facilitator of this success was the support they received from their food service vendors to help navigate food supply challenges that surfaced. For example, the organization that switched entirely to frozen meals during COVID-19 was able to make this change because their food service provider was able to increase the procurement of frozen meals. When expressing appreciation for their food service vendor, a school interviewee stated, “We were just fortunate enough to have a food service provider who wanted to partner, who has the same vision and mind frame that we have to make sure that the students are eating”. 

#### 3.5.7. Community Trust

There were several participating organizations that reported that the community viewed the organization as a trustworthy source from which to receive food. An interviewee from a college that operated a food pantry stated, “Whatever that trust or relationship is that you built with that student over the years, they will accept a bag from you before they go into the grocery store. A lot of times that just eases somebody’s issues with trust”. An afterschool program representative reported that not only did they feel that their clients trusted the organization as a safe place at which to obtain food but the clients also felt they could be open and honest with the organization when they no longer needed food assistance throughout the pandemic. 

#### 3.5.8. Client Satisfaction 

Despite the challenges associated with providing food during the pandemic, most interviewees report that the clients enjoyed the food and/or the program changes that occurred as a result of the pandemic. In an interview with a senior-serving organization, the interviewee stated that the clients not only enjoyed the food they received but also the freedom of the five-pack meals that were distributed once a week as opposed to hot meals daily. This interviewee reported that, according to a survey, many clients wanted to continue the frozen meals. Another interviewee said, “The biggest thing that we heard was about the quality of it. That really stood out to people. … They could tell that it was from scratch and that it was homemade. That really made a big difference to them”. 

### 3.6. Perceived Challenges and Barriers during the COVID-19 Pandemic 

In addition to examining successes, we also examined the barriers and challenges to providing food assistance during the pandemic.

#### 3.6.1. Labor

Due to the increased demand for food, many organizations required additional labor to help prepare, package, and distribute food. To cope with labor challenges, some organizations reported reassigning existing staff to food distribution. This was possible because some staff were involved in operations that ceased during the pandemic and thus were leveraged in food distribution areas. For example, a school representative stated, “We normally have [bus] drivers and then also we have our academic, athletic, and then leadership staff, and everybody was all hands on deck”. However, some organizations had challenges with finding enough paid staff to meet the increased demand for food during the pandemic. As one food bank representative said, “We’re also at a point where it’s work capacity-constrained now...because of the tight driver market. When you look at that, you can only do so much in a day”. 

COVID-19 also changed how organizations worked with volunteers, who were previously a large source of labor for some of the organizations. As described above, some organizations stopped accepting volunteers to decrease COVID-19 exposure. Other organizations continued to accept volunteers but reported a lack of volunteers available. This lack of volunteers led to worry about how to ensure that food was still getting to those who needed it. One food pantry interviewee said, “When the [volunteer] groups started canceling in March, we’re like, ‘Uh-oh’. When you’re doing 20,000 boxes a month, we don’t have [enough] people [working] here to [pack the boxes]”. To cope with a lack of volunteers, some organizations received assistance with labor through partnerships. For example, one food bank reported utilizing the National Guard to pack and distribute food boxes. Some interviewees reported that as the pandemic persisted, volunteers returned. Interviewees expressed how grateful they were for the help volunteers provided and how surprised they were with the large number of volunteers. For example, one interviewee said, “So it has worked for us, especially because of volunteers. The volunteers who said, ‘Hey, I will help you build the boxes. I will help deliver the food’”.

#### 3.6.2. Insufficient Funding and Food Supply

Insufficient funding to purchase food and the materials necessary for packaging food for distribution was reported by a small number of organizations. For example, one organization reported that the amount of food they received from their food bank partners decreased, which necessitated them to purchase food during the pandemic. Although most organizations were able to keep up with the increased demand for food, a few organizations reported they did not have enough food at times. For example, one organization relied on volunteers to go to grocery stores to purchase food for distributions, which was especially needed for foods that were frequently out of stock or had quantity restrictions. A food pantry that serves college students also had challenges obtaining enough food for their pantry. This representative stated one of the biggest challenges was “Just…keeping up with the demand as far as it relates to food. I’m getting ready to order more food because we can’t even keep up with—There’s only so much that we [staff at the college] can donate as humans from our own house”. 

#### 3.6.3. Program Format Change Challenges

Some organizations noted challenges related to changing to a to-go format for their programs. For example, the schools served meals eaten at school pre-pandemic but switched to to-go meals during the pandemic. For families without adequate transportation, this program format change posed a challenge: “If the family doesn’t have transportation, we have people walking up and trying to carry boxes of food a quarter mile, half a mile back home. Especially in winter, that’s infeasible, I think that was our biggest challenge”. Similarly, another organization mentioned the challenges of distributing food outdoors in winter. To make the outdoor distributions possible, they purchased tents and heaters to keep their staff warm.

Other challenges existed when switching to a curbside pick-up model. Since the curbside model was brand new for most organizations, interviewees reported there were many factors that needed to be considered, such as materials to package and distribute food (bags, boxes, to-go containers, etc.) and how perishable items would be kept at appropriate temperatures while outside in the warmer months. A college-serving organization stated, “Getting started up was rough. You had to say, ‘Okay, are we doing boxes? Are we giving bags? Okay, now we got to go buy bags and boxes’. Then you can’t do too heavy because everybody can’t carry it, just those small things were bumps in the road that you had to think about”. 

#### 3.6.4. Audience Gaps

Most commonly, interviewees indicated that people who could not get to food or meal distribution sites were most at risk of not being reached by food programs. This included people without transportation; people with mobility issues, disabilities, or other health issues that prevented them from getting out of their home; and people who did not want to leave their home because of the fear of COVID-19. Seniors were most frequently mentioned as fitting into one or more of these categories. For example, one interviewee stated that seniors were often higher risk because, “in addition to possibly not having transportation, not being able to get out to the grocery store even if they could but they don’t want to, given the fact that the air could potentially be deadly”. 

Another concern regarding who was not being adequately reached was related to geographic regions within the city of Detroit. Interviewees stated that there are pockets in the city that do not receive as much attention as other areas in the city. For instance, in an interview with a food policy and advocacy organization, the interviewee reported that the less-populated areas within the city of Detroit are likely being left out: “People in less populated areas in the city are probably still very hard to reach because where there’s less density, that’s always an issue with resources”.

## 4. Discussion

Through exploring the experiences of 13 local organizations involved in addressing food insecurity, our study found that each of the organizations interviewed made operational and format changes to existing food programs. Additionally, many organizations initiated new food programs to meet the unique challenges that arose during the pandemic. This study identifies valuable information that can be considered in future times of increased need. 

Many programs increased the amount of food distributed during the pandemic. This finding is consistent with the Feeding America 2020 Annual Report that estimated that nearly 5.2 billion meals were distributed by its member food banks in 2020, approximately a 24% increase from the 4.2 billion meals in 2019. Reports from large food banks throughout the U.S. demonstrate similar findings. For instance, in 2020, the Greater Boston Food Bank distributed 94 million pounds of food, and the North Texas Food Bank distributed 35 million pounds of food, a 58% and 84% increase compared to the same time period in 2019, respectively [28,29]. 

Conversely, two school districts reported a decrease in the amount of food provided during the pandemic, which is similar to what occurred nationally. Despite the USDA waivers intended to make school meal distribution easier and to eliminate operational burdens [30], the number of meals served by the National School Lunch Program fell by 33% and meals served by the School Breakfast Program fell by 25% in fiscal year 2020 compared with the prior year [31]. As mentioned by an interviewee, the initiation of the Pandemic-Electronic Benefits Transfer (P-EBT) program offset many families’ need for school meals. These benefits had the convenience of a card, similar to a debit card, that could be used to purchase preferred foods for the entire household from any store accepting EBT [32]. Despite potential interference with school meal participation, the P-EBT has proven to be an effective federal nutrition program for reducing food hardships. During the 2020–2021 school year, in states with higher rates of school closures, the P-EBT reduced child very low food security by 22% and household food insufficiency by 39% [33]. These findings indicate that direct payments through nutrition assistance programs are an effective way to reduce food hardships during emergencies, especially in households with children.

Our study also found that each organization had to make changes to the format and operations of existing food programs. Similar to other studies [20,34], these changes included the transition to pre-packed food or to-go meals for curbside distribution. While making this shift has since proved to be an effective and efficient distribution model, it took away the client-choice model. Like Winkler et al. [22], interviewees expressed the need and desire to return to a client-choice model, particularly so that the selection of food is not determined by the food assistance organization and clients can receive items they prefer and find culturally appropriate. Although not specifically measured in this study, the loss of client-choice models may have disproportionally affected food-insecure households that have special dietary needs. Given that client-choice pantries are overwhelmingly preferred by clients [35] and that it offers users a more dignified experience that can also reduce food waste [36,37,38,39], the need for a charitable feeding system that can address both client preferences and public safety is stressed more than ever during future health crises. 

Some of the perceived challenges reported by organizations provide further information for addressing food insecurity in future crises. Though most of the organizations reported that there was an abundance of food available, some interviewees described food shortages or issues obtaining food. A similar finding was also reported by Larison et al., who found that food procurement at two of its interviewed food pantries was impacted by food shortages and changes in the supply chain [21,40]. While reasons for food shortages varied during the pandemic, consumer panic buying and interruptions in food production areas due to COVID-19 sickness in workers have been identified as contributing factors [18,23,41,42]. Moreover, the cost to make operational changes for COVID-19 put a financial strain on some organizations. Financial challenges were especially true months into the pandemic as funding began to run out. The increased demand for food remained steady in 2020 for most of the organizations in our study. The food supply shortages reported by some interviewees reveal the need for a food supply chain that is effective and adaptable during a crisis. Additionally, they point to the need for adequate funding so that organizations can continue to meet both short- and long-term food needs. 

Similar to other studies [20,23], the organizations in this study also reported labor challenges, especially during the early days of the pandemic. Many interviewees described employees taking on responsibilities outside of their job description, working atypical hours, and attempting to fill in for the lack of volunteers. The lack of volunteers was an unsurprising report since volunteers at food banks are often older adults [43] who are more susceptible to severe COVID-19 infection. Additionally, many food assistance organizations have limited space to social distance, and thus, volunteers may have opted to pause their volunteer work for their own safety. Although the lack of volunteers was a temporary challenge for most of the organizations, it made keeping up with the increased demand for food both challenging and exhausting for staff. Fortunately, each of the organizations reported they were able to adapt to the reduction in volunteers, and like Jablonski et al., some of our organizations reported that the National Guard stepped in to provide support [23]. 

Despite challenges, there were many successes, including organization resilience, adequate funding reported by some organizations, and beneficial collaborations with other organizations. Collaborative efforts are common between food banks and food pantries [44,45], and both existing and new collaborations are vital for addressing food insecurity. COVID-19 safety was also a common success reported by interviewees. Despite challenges obtaining PPE reported by some organizations, most organizations reported implementing the use of masks, gloves, and/or hand sanitation stations, implying good adherence to CDC recommendations [46], and it may have contributed to the community trust reported by some interviewees. Trust that food was being handled safely was especially important since many Americans believed that touching food packaging could transmit COVID-19 [47]. 

A strength of our study was the use of qualitative methods that allowed for an in-depth, detailed understanding of how the organizations involved in addressing food insecurity in Detroit responded to the increase in demand for food during the height of the COVID-19 pandemic and the successes and challenges of their efforts. We also used two independent coders, which allowed multiple perspectives on the data. One limitation of this study is that all interviewees were from formal organizations (nonprofits and public and private education) that existed prior to COVID-19. Thus, food-related efforts by new organizations or informal efforts are not included in this research. Additionally, given the representatives interviewed operate in Detroit, these results may be less generalizable to other geographic regions. Despite limitations, our findings contribute to the body of research dedicated to examining the pandemic’s impact on food programs. 

## 5. Conclusions

This study is among the few existing studies that examine how food programs in the U.S. responded to the COVID-19 pandemic. Nearly all programs changed to a contactless food distribution format, and most were able to increase their distribution of food safely. For programs subject to federal regulations, waivers that eased program rules contributed to their success. However, some organizations experienced challenges with labor, food, or inadequate funding. These findings may be used to plan for how food safety net programs can address food insecurity during future pandemics or other times of increased need. 

## Figures and Tables

**Table 1:** Characteristics of each organization (n = 13) involved in addressing food insecurity and description of the food programs they offered prior to the COVID-19 pandemic.

Organization Type	Food Program(s) Description Pre-Pandemic	Program Audience
Human services nonprofit organization	Prepared meals eaten in a group setting Prepared meals, home-delivered	1 and 2: Seniors (60 years old or older)
Human services nonprofit organization	Client-choice food pantry	Seniors (60 years old or older)
Human services nonprofit organization	Client-choice food pantry	All ages
Food bank	Client-choice pantries (many in collaboration with pantry partners)	All ages
Food bank	Client-choice pantries (many in collaboration with pantry partners) Prepared meals eaten in a group setting (summer only)	All ages Youth under 18 years
Other nonprofit organization: food-policy- and advocacy-focused ^a^	N/A	All ages
Other nonprofit organization: food-waste-reduction-focused ^a^	N/A	All ages
Farmers market	Food market that sells food from local vendors	All ages
College	Client-choice food pantry	College Students
Government agency	Prepared meals eaten in a group setting (summer only)	Youth under 18 years
School district: kindergarten through 12th grade (K-12)	Prepared meals eaten in a group setting (school year)	Students K-12
School district: K-12	Prepared meals eaten in a group setting (school year)	Students K-12
Other nonprofit organization: after school program	Prepared meals eaten in a group setting	8–18 years old

^a^ Organization did not directly distribute food prior to the COVID-19 pandemic.

## Data Availability

The data are not publicly available due to participant confidentiality.

## References

[1] National Research Council (2006). Food Insecurity and Hunger in the United States: An Assessment of the Measure.

[2] Leung C.W., Kullgren J.T., Malani P.N., Singer D.C., Kirch M., Solway E., Wolfson J.A. (2020). Food insecurity is associated with multiple chronic conditions and physical health status among older US adults. Prev. Med. Rep..

[3] Seligman H.K., Laraia B.A., Kushel M.B. (2010). Food Insecurity Is Associated with Chronic Disease among Low-Income NHANES Participants. J. Nutr..

[4] Morales M.E., Berkowitz S.A. (2016). The Relationship between Food Insecurity, Dietary Patterns, and Obesity. Curr. Nutr. Rep..

[5] What is Food Insecurity? Feeding America. https://www.feedingamerica.org/hunger-in-america/food-insecurity.

[6] Ashby S., Kleve S., McKechnie R., Palermo C. (2016). Measurement of the dimensions of food insecurity in developed countries: A systematic literature review. Public Heal. Nutr..

[7] Gebeyehu D.T., East L., Wark S., Islam S. (2022). Impact of COVID-19 on the food security and identifying the compromised food security dimension: A systematic review protocol. PLOS ONE.

[8] Balagtas J., Cooper J. (2021). The Impact of Coronavirus COVID-19 on U.S. Meat and Livestock Markets.

[9] U.S. Bureau of Labor Statistics. Labor Force Statistics from the Current Population Survey. U.S. Bureau of Labor Statistics. https://data.bls.gov/timeseries/LNS14000000.

[10] Mead D., Ransom K., Reed S., Sager S. (2020). The impact of the COVID-19 pandemic on food price indexes and data collection. Mon. Labor Rev..

[11] US Census Bureau. Measuring Household Experiences during the Coronavirus Pandemic Household Pulse Survey. U.S. Census Bureau. Published 2022. https://www.census.gov/data/experimental-data-products/household-pulse-survey.html.

[12] Seligman H.K., Berkowitz S.A. (2019). Aligning Programs and Policies to Support Food Security and Public Health Goals in the United States. Annu. Rev. Public Health.

[13] Feeding America. About Feeding America. Feeding America. https://www.feedingamerica.org/about-us.

[14] Feeding America. The Food Bank Response to COVID in Numbers. Feeding America. Published 2021. https://www.feedingamerica.org/hunger-blog/food-bank-response-covid-numbers.

[15] SNAP COVID-19 Emergency Allotments Guidance. Food and Nutrition Service. https://www.fns.usda.gov/snap/covid-19-emergency-allotments-guidance.

[16] Center on Budget and Policy Priorities (2020). States Are Using Much-Needed Temporary Flexibility in SNAP to Respond to COVID-19 Challenges.

[17] Coleman-Jensen A., Gregory C., Singh A. (2014). Household Food Security in the United States in 2020.

[18] Cranfield J.A.L. (2020). Framing consumer food demand responses in a viral pandemic. Can. J. Agric. Econ. Can. D’agroeconomie.

[19] Louis Aguilar. Detroit Food Banks Overrun by Coronavirus. Bridge Michigan. Published 2020. https://www.bridgemi.com/urban-affairs/detroit-food-banks-overrun-coronavirus-demand.

[20] Castro A.N., White M.A., Ishdorj A., Thompson D., Dave J.M. (2021). The Impact of the COVID-19 Pandemic on Food Distribution at Emergency Food Assistance Organizations in the Southwestern United States: A Qualitative Investigation. Nutrients.

[21] Larison L., Shanks C.B., Webber E., Routh B., Ahmed S. (2021). The Influence of the COVID-19 Pandemic on the Food Supply in the Emergency Food System: A Case Study at 2 Food Pantries. Curr. Dev. Nutr..

[22] Winkler L., Goodell T., Nizamuddin S., Blumenthal S., Atalan-Helicke N. (2022). The COVID-19 pandemic and food assistance organizations’ responses in New York’s Capital District. Agric. Hum. Values.

[23] Jablonski B.B., Casnovsky J., Clark J.K., Cleary R., Feingold B., Freedman D., Gray S., Romeiko X., Olabisi L.S., Torres M. (2021). Emergency Food Provision for Children and Families during the COVID-19 Pandemic: Examples from Five U.S. Cities. Appl. Econ. Perspect. Policy.

[24] Branche-Wilson A., Cooney P. (2020). The Financial Well-Being of Detroit Residents: What Do We Know? Poverty Solutions.

[25] Wilkinson M., Beggin R. Black communities hit harder by coronavirus in Michigan, not just Detroit. Bride Michigan. Published 2020. https://www.bridgemi.com/michigan-health-watch/black-communities-hit-harder-coronavirus-michigan-not-just-detroit.

[26] Rubin H., Rubin I. (2012). Qualitative Interviewing: The Art of Hearing Data.

[27] Miles M., Huberman A.M., Saldana J. (2019). Qualitative Data Analysis: A Methods Sourcebook.

[28] Zack R.M., Weil R., Babbin M., Lynn C.D., Velez D.S., Travis L., Taitelbaum D.J., Fiechtner L. (2021). An Overburdened Charitable Food System: Making the Case for Increased Government Support during the COVID-19 Crisis. Am. J. Public Health.

[29] North Texas Food Bank (2021). North Texas Food Bank 2019–2020 Annual Report.

[30] USDA—FNS Families First Coronavirus Response Act. https://www.fns.usda.gov/resource/families-first-coronavirus-response-act.

[31] Toossi S., Jones J.W., Hodges L. (2021). The Food and Nutrition Assistance Landscape: Fiscal Year 2020 Annual Report.

[32] United States Department of Agriculture USDA ERS—State Guidance on Coronavirus P-EBT. https://www.fns.usda.gov/snap/state-guidance-coronavirus-pandemic-ebt-pebt.

[33] Schanzenbach D.W., Bauer L., Ruffini K. (2021). An Update on the Effect of Pandemic EBT on Measures of Food Hardship. Brookings Institution. https://www.brookings.edu/research/an-update-on-the-effect-of-pandemic-ebt-on-measures-of-food-hardship/.

[34] Kinney S., Simon C., Caspi C., Schwartz M. (2021). Under Pressure: Prioritizing Healthy Hunger Relief during the Covid-19 Pandemic.

[35] Caspi C.E., Davey C., Barsness C.B., Gordon N., Bohen L., Canterbury M., Peterson H., Pratt R. (2021). Needs and Preferences among Food Pantry Clients. Prev. Chronic Dis..

[36] Remley D.T., Zubieta A.C., Lambea M.C., Quinonez H.M., Taylor C. (2010). Spanish- and English-Speaking Client Perceptions of Choice Food Pantries. J. Hunger. Environ. Nutr..

[37] Arnold J.M. Charity Food Programs That Can End Hunger in America. Second Harvest Gleaners Food Bank of West Michigan; 2004. https://www.endhungerinamerica.org/publications/charity-food-programs-that-can-end-hunger-in-america/chapter-nine-reduce-waste-and-humiliation-let-clients-assemble-their-own-food-boxes/.

[38] Henne B. Client-Choice Food Pantry Model Reduces Food Waste and Improves Food Distribution. MSU Extension. Published 2013. https://www.canr.msu.edu/news/client_choice_food_pantry_model_food_waste_improves_food_distribution.

[39] Pruden B., Poirier L., Gunen B., Park R., Hinman S., Daniel L., Gu Y., Katragadda N., Weiss J., Gittelsohn J. (2020). Client Choice Distribution Model Is Associated with Less Leftover Food in Urban Food Pantries. Curr. Dev. Nutr..

[40] Greer A.E., Cross-Denny B., McCabe M., Castrogivanni B. (2016). Giving Economically Disadvantaged, Minority Food Pantry Patrons’ a Voice: Implications for Equitable Access to Sufficient, Nutritious Food. Fam. Community Health.

[41] Nicola M., Alsafi Z., Sohrabi C., Kerwan A., Al-Jabir A., Iosifidis C., Agha M., Agha R. (2020). The socio-economic implications of the coronavirus pandemic (COVID-19): A review. Int. J. Surg..

[42] Ben-Hassen T., El-Bilali H., Allahyari M.S. (2020). Impact of COVID-19 on Food Behavior and Consumption in Qatar. Sustainability.

[43] Federal Emergency Management Agency (2020). Food Bank Worker, Volunteer, and Client Safety during COVID-19.

[44] Guo C., Acar M. (2005). Understanding Collaboration among Nonprofit Organizations: Combining Resource Dependency, Institutional, and Network Perspectives. Nonprofit Volunt. Sect. Q..

[45] Parker M.A., Mook L., Kao C.-Y., Murdock A. (2020). Accountability and Relationship-Definition among Food Banks Partnerships. Voluntas.

[46] United States Department of Homeland Security COVID-19 Best Practice Information: Food Banks. 2020. p. 5. https://www.fema.gov/sites/default/files/2020-07/fema_covid_bp-food-bank-coordination.pdf.

[47] Niles M.T., Bertmann F., Morgan E.H., Wentworth T., Biehl E., Neff R. Food Access and Security during Coronavirus: A Vermont Study; College of Agriculture and Life Sciences Faculty Publications. https://scholarworks.uvm.edu/calsfac/21/.

